# Genetic Variants in the *EPCAM* Gene Is Associated with the Prognosis of Transarterial Chemoembolization Treated Hepatocellular Carcinoma with Portal Vein Tumor Thrombus

**DOI:** 10.1371/journal.pone.0093416

**Published:** 2014-04-09

**Authors:** Xiaohe Yu, Naijian Ge, Xu Guo, Shuqun Shen, Jun Liang, Xiaojun Huang, Shaogui Wan, Jingliang Xing, Qichao Huang, Yefa Yang

**Affiliations:** 1 Department of Radioactive Intervention, Eastern Hepatobiliary Surgery Hospital, Second Military Medical University, Shanghai, China; 2 State Key Laboratory of Cancer Biology, Cell Engineering Research Center and Department of Cell Biology, Xi’an, Shaanxi, China; 3 Experimental Teaching Center of Basic Medicine, Fourth Military Medical University, Xi’an, Shaanxi, China; 4 Pharmaceutical College, Henan University, Kaifeng, Henan, China; University of Pisa, Italy

## Abstract

The epithelial cell adhesion molecule (EPCAM) is involved in the tumorigenesis and progression of many malignancies, including hepatocellular carcinoma (HCC). Single nucleotide polymorphisms (SNPs) of *EPCAM* have been reported to be with the risk and prognosis of several malignancies. However, the association of SNPs in *EPCAM* gene with the prognosis of HCC patients has never been investigated. In this study, two functional SNPs (rs1126497 and rs1421) in the *EPCAM* gene were selected and genotyped in a cohort of 448 unresectable Chinese HCC patients treated by TACE. The association of the two SNPs with the overall survival (OS) of patients was assessed by univariate and multivariate Cox proportional hazards model and Kaplan-Meier curve. Our data showed that there was no significant association between either SNP and OS of patients. However, in the stratified analysis, the variant-containing genotypes (WV+VV) of SNP rs1126497 exhibited a significant association with poorer OS in HCC patients who had portal vein tumor thrombus (PVTT) in multivariate analysis of Cox proportional hazard model (hazard ratio, 1.71; 95% confidence interval, 1.16–2.53, *P* = 0.007), and in Kaplan-Meier curve analysis (*P* = 0.023), comparing to those carrying wild-type genotype. Our results suggest that SNP rs1126497 in the *EPCAM* gene may serve as an independent prognosis biomarker for unresectable HCC patient with PVTT, which warranted further validating investigation.

## Introduction

Hepatocellular carcinoma (HCC) is one of the most prevalent malignancies worldwide, and its morbidity and mortality rates have escalated in recent years[1]. Despite improvements in surveillance and clinical treatment strategies, the prognosis of HCC patients remains poor. One major reason is that most HCC patients are diagnosed at intermediate to advanced stages, and thus curative therapies such as resection, transplantation, or percutaneous ablation are not suitable[2]. Transarterial chemoembolization (TACE) is the most widely-used treatment for unresectable HCC and is often recommended as the first-line therapy for HCC patients at intermediate stage of the disease[3], [4]. However, the prognosis for HCC patients treated by TACE is greatly varied according to disease status. For example, cohort studies with long-term follow-ups have showed a median survival time of 20 months for patients with HCC at intermediate stages and 12 months for patients at advanced stages with portal vein invasion[5]. Traditional clinicopathological parameters such as tumor morphology, histopathological features, concentration of serum alpha fetoprotein (AFP) and tumor stage offer limited information for prognosis prediction and fail to guide the therapeutic schedule for individual patient. Therefore, it is extremely urgent to explore novel biomarkers to discriminate patient groups with different clinical outcomes and direct the treatment for HCC patients.

Epithelial cell adhesion/activating molecule (EPCAM) is a 30–40 kDa type I membrane protein of 314 amino acids[6]. Besides cell adhesion, EPCAM is also involved in other biological functions including signal transduction, cell proliferation, differentiation and tissue regeneration[7]. Osta and colleagues have reported that down-regulation of EPCAM by siRNA inhibits cell proliferation and migration [8]. Recent studies have revealed that EPCAM is over-expressed in a variety of human cancers, including lung, esophagus, gastric, breast, colorectal, and hepatocellular carcinomas[9]. Overexpression of EPCAM is associated with high proliferation and invasive activity in tumor cells as well as with poorer survival in cancer patients[10]. Additionally, EPCAM has been widely explored as cancer biomarker in experimental and observational studies[11]. A recent study has identified that EPCAM-positive cells from whole blood have stem cell-like characteristics and are associated with poor prognosis in HCC patients[12].

Single nucleotide polymorphisms (SNPs) represent the most common form of genetic diversity within a species and account for much of the variation in genetic traits between patients [13], including disease susceptibility, prognosis and response to therapy. In addition to amino acid change, SNPs directly affect gene functions through various translational or post-translational mechanisms, such as altering miRNA binding, protein folding, the spliceosome formation or mRNA stability [14]. Jiang et al have reported that a non-synonymous polymorphism Thr115Met (C/T in SNP rs1126497)in the *EPCAM* gene is associated with an increased risk of breast cancer and cervical cancer[15], [16]. Furthermore, our previous study has demonstrated that SNP rs1126497 is significantly associated with the survival of non small cell lung cancer patients[17]. These findings suggest that SNPs in the *EPCAM* gene may play an important role in the initiation and progression of cancer. However, to date, the association between genetic variants in *EPCAM* gene and clinical outcome has not been investigated in HCC patients.

In this study, we examined the genotype in two functional SNP loci (rs1126497 and rs1421) in *EPCAM* gene and assessed the associations of the two SNPs with the overall survival (OS) in a Chinese cohort of 448 unresectable HCC patients treated by TACE. To the best of our knowledge, this is the first study to investigate the prognostic role of *EPCAM* gene polymorphisms in HCC.

## Materials and Methods

### Patient Population

A total of 493 Han Chinese patients with unresectable HCC were recruited at the Department of Radioactive Intervention of Eastern Hepatobiliary Surgery Hospital, Second Military Medical University in Shanghai, China between February 2008 and December 2011. All patients had no previous history of other cancers or cancer-related treatment at enrollment, and were newly diagnosed as HCC by imaging technologies. TACE was used as the first-line treatment for all patients. TACE treatment was applied as previously described [18]. 45 cases were excluded, including 32 patients who had incomplete clinical data or failed follow up, and 13 with poor quality of DNA. Finally, 448 patients were included and successfully genotyped. Detailed clinical information was collected through medical record review by treating physicians A standard follow-up was performed by a trained clinical specialist through on-site interview, direct calling, or medical record review. The latest follow-up data in this analysis were obtained in June 2013. OS was defined as the interval from the first TACE treatment to the date of death or last follow-up. This study was approved by the Ethic Committee of the Second Military Medical University, and signed informed consent was obtained from each participant.

### SNP Selection and Genotyping

Five milliliter of venous blood sample was collected from each patient before any treatment. Leukocyte genomic DNA was extracted from blood samples using the E.Z.N.A. Blood DNA Midi Kit (Omega Bio-Tek, Norcross, GA). Functional SNPs in EPCAM gene was selected using a set of Web-based SNP selection tools (http://snpinfo.niehs.nih.gov/snpinfo/snpfunc.htm) based on linkage disequilibrium and predicted functional characteristics of both coding and noncoding SNPs. Same as previously reported [15], two functional SNPs in *EPCAM* gene, rs1126497 and rs1421, with >5%minor allele frequency in Asian population were selected for genotype assay. rs1126497 (C/T) is a non-synonymous polymorphism in exon 3 of EPCAM gene, leading to a transition from 115 Met to 115 Thr which may induce alteration of EPCAM structure and consequently function of the protein. The A/G polymorphism of rs1421 is in the 3′UTR of EPCAM. The conversion from A to G of rs1421 polymorphism may cause loss of a has-miR-1183 binding site and cause new combination of micro-RNA has-miR-370 and has-miR-517a, which may affect the translation of EPCAM mRNA and possibly be influential to function of EPCAM. Genotyping was carried out on the iPLEX genotyping system (Sequenom, San Diego, CA). Laboratory persons who conducted genotyping assays were blinded to patient information. The average call rate for the SNP assays was 99.3%. Strict quality controls were implemented during genotyping, with >99.0% concordance.

### Statistical Analysis

The SPSS Statistics 19.0 software (IBM) was used for all statistical analyses. Continuous variables including age, tumor size and serum AFP concentration were transformed into categorical variables in all regression analyses. Univariate and multivariate Cox regression analyses to explore prognostic factors for OS. In the multivariate analyses, the association between single SNP and OS was estimated as hazard ratios (HRs) with adjusting for age, gender, HBsAg status, serum AFP, tumor size, BCLC stage and TACE treatment number where appropriate. The association between SNPs and OS was assessed in additive, dominant and recessive models, and dominant model was chose for further analysis. To exclude the effects of confounding factors, the association between SNPs in *EPCAM* gene and OS of HCC was assessed in multivariate Cox regression model stratified by each clinical characteristic factor with adjustment for the remaining factors. Kaplan-Meier curve and log-rank test were used to assess the differences of OS among different subgroups. Logistic regression was used to assess the association of SNPs with and risk of PVTT, which adjusted by age, gender, HBsAg, serum AFP, tumor size and number of tumor. All tests were two-sided, and α* = *0.05 was considered as the threshold of statistical significance.

## Results

### Clinical Characteristics and their Association with the OS of HCC Patients

Clinical characteristics of the 448 unresectable HCC patients with first-line TACE treatment are summarized in Table 1. The median age at the time of diagnosis was 54 years (range, 27–82 years) for all patients. A majority of patients (391, 87.3%) were male. There were 63 (14.7%) patients with negative serum HBsAg, while there were 205 (45.8%) patients who had serum AFP≤200 ng/ml. The majority of patients (70.1%) had a tumor size larger than 5 cm, while58.9% had multiple lesions. At diagnosis, 134 (29.9%) patients were present with PVTT. BCLC stage status was as follows: 221 (49.3%) patients at B stage, and 227 (50.7%) at C stage. Finally, the patient cohort received a total of 1873 TACE procedures (mean 4 per patient, range 1–7). During the median follow-up of 30.0 months (ranging from 2.4 to 72.8 months), 339 patients died of HCC.

**Table 1:** Distributions of clinical characteristics and multivariant Cox regression analysis of prognostic factors in HCC patients**.**

Variables	No. of total patients (%)	No. of death (%)	Univariate analysis		Multivariate analysis^a^	
			HR (95%CI)	*P* value	HR (95%CI)	*P* value
**Age**						
≤54	236 (52.7%)	179 (52.8%)	Ref.		Ref.	
>54	212 (47.3%)	160 (47.2%)	0.86 (0.69–1.06)	0.169	0.58 (0.28–1.21)	0.147
**Gender**						
Female	57 (12.7%)	40 (11.8%)	Ref.		Ref.	
Male	391 (87.3%)	299 (88.2%)	1.16 (0.83–1.62)	0.373	1.25 (0.90–1.76)	0.188
**HBsAg**						
No	65 (14.5%)	48 (14.2%)	Ref.		Ref.	
Yes	383 (85.5%)	291 (85.8%)	1.12 (0.82–1.52)	0.468	1.03 (0.74–1.43)	0.853
**Serum AFP**						
≤200	205 (45.8%)	140 (41.3%)	Ref.		Ref.	
>200	243 (54.2%)	199 (58.7%)	1.63 (1.31–2.03)	<0.0001	1.35 (1.08–1.70)	0.010
**Tumor size (cm)**						
≤5	134 (29.9%)	80 (23.6%)	Ref.		Ref.	
>5	314 (70.1%)	259 (76.4%)	2.53 (1.96–3.27)	<0.0001	1.95 (1.46–2.61)	<0.0001
**Number of lesions**						
Single	184 (41.1%)	128 (37.8%)	Ref.		Ref.	
Multiple	264 (58.9%)	211 (62.2%)	1.40 (1.12–1.75)	0.003	1.07 (0.83–1.37)	0.606
**PVTT**						
No	314 (70.1%)	221 (65.2%)	Ref.		Ref.	
Yes	134 (29.9%)	118 (34.8%)	2.43 (1.93–3.05)	<0.0001	1.82 (1.42–2.33)	<0.0001
**BCLC stage**						
B stage	221 (49.3%)	142 (41.9%)	Ref.		Ref.	
C stage	227 (50.7%)	197 (58.1%)	2.18 (1.75–2.71)	<0.0001	1.54 (1.20–1.98)	0.001
**Number of TACE**						
≤4	200 (44.6%)	146 (43.1%)	Ref.		Ref.	
>4	248 (55.4%)	193 (56.9%)	1.04 (0.83–1.28)	0.753	1.79 (0.85–3.74)	0.125

Abbreviations: CI, confidence interval; HR, hazard ratio; Ref., reference.

a Adjusted for age, gender, HBsAg, serum AFP, tumor size, BCLC stage and number of TACE where appropriate.

Univariate Cox regression analyses showed a significant poorer overall survival in patients with high level of serum AFP (HR, 1.63; 95% CI, 1.31–2.03, *P*<0.0001), in patients with larger size of tumor (HR, 2.53; 95% CI, 1.96–3.27; *P*<0.0001), in patients with multiple lesions (HR, 1.40; 95% CI, 1.12–1.75; *P* = 0.003), in patients with higher degree of BCLC stage (HR, 2.18; 95% CI, 1.75–2.71, *P*<0.0001), and in patients with existence of PVTT (HR, 2.43; 95% CI, 1.93–3.05; *P*<0.0001) when compared with corresponding control patients groups. Furthermore, in multivariate Cox regression analysis, significantly increased death risk was still observed in patients with high level of serum AFP (HR, 1.35; 95% CI, 1.08–1.70; *P* = 0.010), in patients with large size of tumor (HR,1.95; 95% CI 1.46–2.61, *P*<0.0001), in patients with existence of PVTT(HR, 1.82; 95% CI 1.42–2.33, *P*<0.0001) and in patients with high BCLC stage (HR, 1.54; 95% CI 1.20–1.98, *P* = 0.001).

### Prognostic Significance of SNPs in *EPCAM* Gene in HCC Patients Treated with TACE

**Figure 1:**
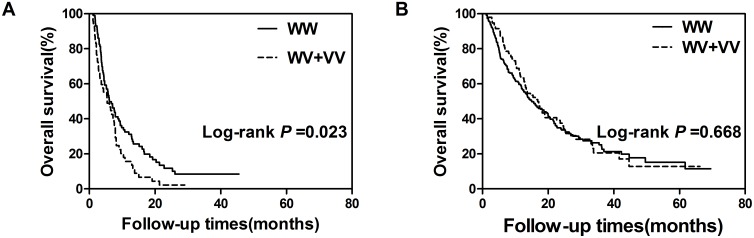
Kaplan-Meier plots of overall survival in patients without PVTT (A) and in patients with PVTT (B).

No significant association between either of the two SNPs and the OS of HCC patients was observed in univariate and multivariate Cox regression analysis (Table 2). We then conducted the stratified analysis based on age, serum AFP, tumor size, number of tumor lesions, PVTT status, BCLC stage and number of TACE treatment. Interestingly, we identified a significant association between variant-containing genotype of rs1126497 and poor OS in patients with PVTT (HR = 1.71; 95% CI, 1.16–2.53, *P* = 0.007) (Table 3). Similarly, Kaplan-Meier curve analysis showed a significantly shorter median survival time in patients with variant-containing genotype of rs1126497 than those carrying wild-type genotype in patients with PVTT (Log rank *P* = 0.023, Figure 1). However, SNP rs1421 was not associated with HCC patients in any stratified subgroups.

**Table 2:** Association of SNPs with clinical outcome of HCC patients.

			Univariate analysis		Multivariate analysis^a^	
SNP	Genotype	Death/Total	HR (95%CI)	*P* value	HR (95%CI)	*P* value
rs1126497	WW	229/307				
	WV	98/126	1.08 (0.85–1.37)	0.517	1.13 (0.88–1.44)	0.342
	VV	12/15	1.29 (0.72–2.32)	0.385	1.36 (0.75–2.46)	0.299
	Additive		1.10 (0.91–1.34)	0.331	1.14 (0.94–1.39)	0.190
	Dominant		1.10 (0.88–1.38)	0.406	1.15 (0.91–1.44)	0.244
	Recessive		1.27 (0.71–2.25)	0.425	1.32 (0.74–2.36)	0.354
rs1421	WW	237/306				
	WV	95/132	0.92 (0.72–1.17)	0.486	0.89 (0.71–1.14)	0.331
	VV	7/10	0.83 (0.39–1.76)	0.623	0.86 (0.39–1.81)	0.668
	Additive		0.92 (0.74–1.13)	0.413	0.90 (0.73–1.11)	0.331
	Dominant		0.91 (0.72–1.15)	0.437	0.89 (0.70–1.12)	0.322
	Recessive		0.85 (0.40–1.80)	0.669	0.89 (0.42–1.90)	0.767

a Multivariate analyses were adjusted for age, gender, HBsAg, serum AFP, tumor size, BCLC stage and number of TACE.

**Table 3:** Stratified analysis of association between single SNPs and overall survival in HCC Patients.

		rs1126497			rs1421		
Strata	Genotype	Death/Total	HR (95%CI)^a^	*P* value	Death/Total	HR (95%CI)^a^	*P* value
Age (year)							
≤54	WW	124/163	Ref.		122/154	Ref.	
	WV+VV	55/73	1.13(0.81–1.56)	0.475	57/82	0.77(0.56–1.05)	0.100
>54	WW	105/144	Ref.		115/152	Ref.	
	WV+VV	55/68	1.13(0.81–1.59)	0.462	45/60	1.09(0.75–1.57)	0.647
Serum AFP (ng/ml)							
≤200	WW	96/143	Ref.		98/134	Ref.	
	WV+VV	44/62	1.21(0.84–1.73)	0.302	42/71	0.72(0.49–1.04)	0.077
>200	WW	133/164	Ref.		139/172	Ref.	
	WV+VV	66/79	1.09(0.81–1.47)	0.581	60/71	1.16(0.84–1.60)	0.371
Tumor size (cm)							
≤5	WW	48/87	Ref.		57/95	Ref.	
	WV+VV	32/47	1.36(0.87–2.15)	0.181	23/39	1.34(0.79–2.27)	0.284
>5	WW	181/220	Ref.		180/211	Ref.	
	WV+VV	78/94	1.03(0.79–1.35)	0.824	79/103	0.84(0.64–1.10)	0.205
Number of lesions							
Single	WW	91/128	Ref.		91/127	Ref.	
	WV+VV	37/56	0.99(0.67–1.48)	0.964	37/57	0.90(0.61–1.35)	0.624
Multiple	WW	138/179	Ref.		146/179	Ref.	
	WV+VV	73/85	1.22(0.91–1.64)	0.186	65/85	0.89(0.66–1.20)	0.459
PVTT							
No	WW	156/220	Ref.		157/217	Ref.	
	WV+VV	65/94	0.94(0.70–1.26)	0.691	64/97	0.98(0.73–1.33)	0.912
Yes	WW	73/87	Ref.		80/89	Ref.	
	**WV+VV**	**45/47**	1.71(1.16–2.53)	0.007	38/45	0.87(0.58–1.30)	0.493
BCLC stage							
B stage	WW	95/150	Ref.		97/149	Ref.	
	WV+VV	47/71	1.07(0.75–1.53)	0.718	45/72	1.13(0.77–1.65)	0.546
C stage	WW	134/157	Ref.		140/157	Ref.	
	WV+VV	63/70	1.16(0.86–1.58)	0.331	57/70	0.81(0.59–1.11)	0.191
**Number of TACE**							
≤4	WW	122/160	Ref.		117/149	Ref.	
	WV+VV	54/72	1.08(0.78–1.51)	0.638	59/83	0.83(0.61–1.15)	0.260
>4	WW	107/147	Ref.		120/157	Ref.	
	WV+VV	56/69	1.15(0.82–1.59)	0.423	43/59	1.00(0.69–1.45)	0.992

a In each stratified analysis, HRs were adjusted for age, gender, HBsAg, serum AFP, tumor size, BCLC stage and number of TACE except for the stratifier.

### Association of SNPs with Formation of PVTT

Since the significant association result between SNPs and cancer outcomes was observed in HCC patients with PVTT, we further examined whether the genotypes of these SNPs have effect on the formation of PVTT by logistic regression analysis with adjustment for age, gender, HBsAg, serum AFP, tumor size and number of tumor. As shown in Table 4, we failed to find a significant association between the genotypes of rs1126497 or rs1421 and the formation of PVTT.

**Table 4:** Association of SNPs with formation of PVTT.

SNP	Genotype	Patients without PVTT	Patients with PVTT	OR (95% CI)^a^	*P* value
rs1126497	WW	220	87	Ref.	
	WV	84	42	1.39 (0.86–2.25)	0.175
	VV	10	5	1.72 (0.53–5.55)	0.367
	WV+VV	94	47	1.42 (0.90–2.26)	0.134
rs1421	WW	217	89	Ref.	
	WV	91	41	1.01 (0.63–1.63)	0.960
	VV	6	4	1.95 (0.48–7.90)	0.348
	WV+VV	97	45	1.06 (0.67–1.68)	0.801

a Adjusted for age, gender, HBsAg, serum AFP, tumor size, number of lesions.

## Discussion

In the present study, we assessed the effects of 2 functional SNPs (rs1126497 and rs1421) in the *EPCAM* gene on the OS of a cohort of unresectable Chinese HCC patients treated by TACE. We found that patients carrying the homozygous variant (VV) genotype and heterozygous variant (WV) of SNP (rs1126497) had significantly poorer OS than those carrying the wild genotype.

HCC patients with PVTT have an extremely poor prognosis. Therefore, it is important to evaluate potential prognostic factors for the elucidation of treatment strategies. TACE, radiation, systematic chemotherapy, and targeted therapy with sorafenib were all used for treatment of patients with PVTT[19], [20]. However, the most effective treatment strategy for HCC with PVTT remains to be established. Recently, a perspective study has demonstrated the survival benefit of TACE in Chinese patients with unresectable HCC and with PVTT[21]. Subsequently, a meta-analysis also indicated that TACE treatment resultedin survival benefit for advanced HCC with PVTT, even with main portal vein obstruction[22]. The responses to TACE treatment varies among patients with different characteristics. Therefore, it is important to choose adequate therapeutics based on prognostic factors, including imaging, clinical and molecular data. Several clinical parameters have been identified as prognostic factors of HCC patients with PVTT [23]. However, until now, few biomarkers have been identified to predict the prognosis and responses to treatment of HCC patients with PVTT. In our study, we found that that SNP rs1126497 in the *EPCAM* gene was significantly associated with the outcome of HCC patients with PVTT. These findings suggest that rs126497 may serve as an biomarker to distinguish patients with different prognosis and thus would help to improve the individualized management of HCC patients with PVTT.

EPCAM plays an important role in the initiation and progression of cancer. A series studies have reported that EPCAM promotes tumor formation and metastasis either by disrupting the link between a-catenin and F-actin [24] or acting as a signal transducer after sequential cleavage by tumor necrosis-factor alpha-converting enzyme (TACE/ADAM17) and a gamma-secretase complex containing presenilin 2 (PS-2) [25]. EPCAM can also contribute to tumorigenesis and metastasis by facilitating the immune escape of tumor cells[26]. Previous studies have showed that overexpression of EPCAM is significantly associated with the poor clinical outcome of HCC, and EPCAM-positive cancer patients commonly exhibit an advanced tumor stage[27]. In addition, EPCAM has recently been identified to be a surface biomarker of cancer stem cells (CSCs), which refer to a subset of phenotypically distinct cells mainly responsible for tumor growth and heterogeneity maintenance [28]. Taro Yamashita et al have reported that EPCAM-positive HCC cells display several liver cancer stem cell-like traits including the abilities to self-renew and differentiate[29]. These findings indicate that EPCAM may serve as an potential target for the treatment of cancer.

SNP may affect the expression and functions of genes. Previous studies have suggested the contribution of polymorphisms in *EPCAM* gene to the increased risk of breast cancer and cervical cancer[15], [16], and our recent study also showed that non-synonymous SNP rs1126497 in *EPCAM* gene may be a potential prognostic marker for NSCLC patients[17]. In consistence with these findings, we found that SNP rs1126497 is associated with the OS of HCC patients with PVTT. However, little is known to date about the association of polymorphisms in *EPCAM* gene with prognosis of HCC patients. Online analysis indicate that the C/T polymorphism (rs1126497) in exon 3 of *EPCAM* gene leads to a transition from Met115Thr variation, which may induce alteration of EPCAM structure and consequently function of the protein, while the A/G polymorphism of rs1421 in the 3′UTR of *EPCAM* gene may cause the loss of a has-miR-1183 binding site, which may affect the protein expression level of EPCAM [17]. However, SNP rs1421 is not associated with either the risk or the prognosis of cancers. In our study, we also found that SNP rs1421 is not associated with the prognosis of HCC. In addition, SNP rs1126497 is only associated with the prognosis of HCC patients with PVTT but not those without PVTT. These data collectively suggest that the biological role of SNP in the development of cancer may display a disease-specific manner. Further functional studies are needed to elucidate the effects of the genetic variants on the functions of EPCAM in the development of cancer.

Our study has several limitations. First, because it was a retrospective study, potential selection bias in patient enrollment could not be ruled out. Second, since our study population was restricted to Han Chinese, further evaluation of our results in populations with other ethnicities is needed to generalize the clinical uses of our findings. Third, we could not rule out the potential false positive results in the multiple and subgroup testing. Therefore, future prospective studies with comprehensive statistical analyses are warranted to further validate our findings.

In conclusion, our results suggest that SNP rs1126497 in *EPCAM* gene combined with multiple tumor or PVTT can better predict prognosis for unresectable HCC patient treated with TACE. Our findings contribute to the current understanding on the functional roles of *EPCAM* gene SNPs in clinical outcome of HCC patients. To the best of our knowledge, this is the first report on the association of *EPCAM* gene SNPs with clinical outcome of HCC patients.
